# Jasmonate signaling drives defense responses against *Alternaria alternata* in chrysanthemum

**DOI:** 10.1186/s12864-023-09671-0

**Published:** 2023-09-19

**Authors:** Shuhuan Zhang, Weihao Miao, Ye Liu, Jiafu Jiang, Sumei Chen, Fadi Chen, Zhiyong Guan

**Affiliations:** 1 State Key Laboratory of Crop Genetics & Germplasm Enhancement and Utilization, Key Laboratory of Landscaping, Ministry of Agriculture and Rural Affairs, Key Laboratory of State Forestry and Grassland Administration On Biology of Ornamental Plants in East China, College of Horticulture, Nanjing Agricultural University, Nanjing, 210095 China; 2 Zhongshan Biological Breeding Laboratory, No.50 Zhongling Street, Nanjing, 210014 Jiangsu China

**Keywords:** JA signaling, *Alternaria alternata*, *Chrysanthemum morifolium*, Defense responses

## Abstract

**Background:**

Black spot disease caused by the necrotrophic fungus *Alternaria* spp. is one of the most devastating diseases affecting *Chrysanthemum morifolium*. There is currently no effective way to prevent chrysanthemum black spot.

**Results:**

We revealed that pre-treatment of chrysanthemum leaves with the methy jasmonate (MeJA) significantly reduces their susceptibility to *Alternaria alternata*. To understand how MeJA treatment induces resistance, we monitored the dynamics of metabolites and the transcriptome in leaves after MeJA treatment following *A. alternata* infection. JA signaling affected the resistance of plants to pathogens through cell wall modification, Ca^2+^ regulation, reactive oxygen species (ROS) regulation, mitogen‐activated protein kinase cascade and hormonal signaling processes, and the accumulation of anti-fungal and anti-oxidant metabolites. Furthermore, the expression of genes associated with these functions was verified by reverse transcription quantitative PCR and transgenic assays.

**Conclusion:**

Our findings indicate that MeJA pre-treatment could be a potential orchestrator of a broad-spectrum defense response that may help establish an ecologically friendly pest control strategy and offer a promising way of priming plants to induce defense responses against *A. alternata*.

**Supplementary Information:**

The online version contains supplementary material available at 10.1186/s12864-023-09671-0.

## Background

Plants possess innate immune systems that rely on a broad range of constitutive, inducible anti-fungal molecules and a large-scale transcriptional reprogramming in the host plant that is activated via a complex signaling network. Initially, pathogens must overcome the plant's physical barriers, such as the waxy cuticle and the cell wall, which leads to cell wall damage [1, 2]. Pathogen-associated molecular patterns (PAMPs) are recognized by plant cell-surface pattern-recognition receptors (PRRs) that induce signals through plasma-membrane-associated co-receptor kinases and intracellular protein kinases. Through the activation of NADPH oxidases encoded by respiratory burst oxidase homologue (RBOH) genes, mitogen-activated protein kinases (MAPKs), and the induction of downstream cellular immune responses, ligand-dependent association between PRRs and protein kinases causes an influx of Ca^2+^ and the production of ROS [3]. The defense response can be directly induced by gene expression and indirectly by the stimulation and fine-tuning of hormones such as jasmonic acid (JA), salicylic acid (SA), and ethylene (ET) [4, 5]. Previous research has shown that SA is a major hormone against biotrophic and hemi-biotrophic pathogens, which rely on living plant tissue for nutrients [6–8]. In contrast, the JA/ET pathway is critical for plant defense against necrotrophic diseases [9, 10].

JA biosynthesis begins with the release of α-linolenic acid from membrane lipids in the chloroplast [11, 12]. Subsequently, the bioactive hormone jasmonoyl-isoleucine (JA-Ile) is created when JA is conjugated to isoleucine. Moreover, inductive signals like PAMPs are recognized by PRRs at the cell surface to trigger de novo synthesis of JA-Ile from plastid lipids. JA-Ile acts as the major bioactive JA to activate core JA signaling by binding with its coreceptor, the Skp1-Cullin1-F-box-type (SCF) protein ubiquitin ligase complex SCF^COI1^-JAZ [13]. At present, many studies have reported that exogenous feeding or external stimuli, can induce endogenous JA synthesis and signal transduction to activate JA signaling [14, 15]. Thereafter, JA signaling activates multiple downstream signaling pathways and transcription factors (TFs) to affect cell wall modification, the production of pathogenesis-related proteins, and the accumulation of anti-fungal molecules that regulate resistance to necrotrophic pathogens and associated stress responses [16–18]. Generally, many defense secondary metabolites, such as phenylpropanoids, flavonoids, and phytoalexins, which serve as signal molecules in plant–pathogen interactions, have anti-fungal or anti-oxidant characteristics [19–21].

Several studies have explored the mechanism of host–pathogen interactions for *Alternaria alternata*. For example, in *Nicotiana attenuate*, JA signaling regulates the biosynthesis and accumulation of phytoalexin scopoletin through MYC2, and activated abscisic acid (ABA) signaling promotes stomatal closure to enhance resistance to *A. alternata* [22]. The latest evidence suggests that NaWRKY3 is an important factor in scopoletin synthesis, which transcriptionally regulates Rboh-mediated stomatal closure [23]. In apple (*Malus domestica* Borkh.), ET, JA, and SA signaling and pathogen-induced release of elicitors from the cell wall also contribute to *A. alternata* resistance [24]. In chrysanthemum, the cross-talk between hormone and Ca^2+^ signal transduction pathways is the most effective defense response against *A. alternata* infection [25, 26]. Moreover, the transgenic silencing of the Mildew Resistance Locus O gene (*CmMLO17*) in chrysanthemum regulates ABA and Ca^2+^ signaling pathways, resulting in reduced susceptibility to *A. alternata* infection [27].

*C. morifolium* is an important member of Asteracese that has ornamental, medical, and edible value. However, both the quality and quantity of chrysanthemum are severely affected by fungal diseases. *A. alternata* is a necrotrophic fungus that is ubiquitously found on various plant species. It causes a black spot disease that severely affects chrysanthemum cultivation. The disease usually occurs in mature leaves [28, 29], and once established, it can spread rapidly through plant residue, soil, and atmosphere, especially in warm and humid environmental conditions. In the early stage of the infection, *A. alternata* damages plant tissues by secreting toxins and cell wall degrading enzymes (CWDEs) to help pathogens invade. Then, pathogen obtains nutrients from decaying tissue, and lead to round spots with dark mildew layer. Finally, premature aging and even death were occurred on the plant. The widespread occurrence of this disease causes economic losses and hinders crop production. Broad spectrum fungicides are currently less effective against this disease, cause serious environmental pollution, and have a high cost and energy consumption. Therefore, it is necessary for elicitors or organic compounds to promote resistance by triggering the host's defense mechanism against infections [30].

Using transcriptomic analysis, we reported that *A. alternata* activates the transcription of JA biosynthesis and signaling genes in chrysanthemum [31]. However, the molecular mechanism associated with JA-induced defense responses against *A. alternata* in chrysanthemum is largely unknown. Here, we revealed that MeJA pre-treatment of the chrysanthemum significantly reduced its susceptibility to *A. alternata* infection. By keeping track of the large-scale metabolomic and transcriptomic changes in the leaves after MeJA pre-treatment and *A. alternata* infection, we pinpointed potential defense response underpinning mechanisms. We also indicated that MeJA treatment has a little effect on gene expression but does induce regulatory genes associated with the defense response such as receptor kinases, Ca^2+^ regulation, TFs, and phytohormones. When leaves were pretreated with MeJA before infection with *A. alternata*, we noticed transcriptional reprogramming of genes related to the cell wall, resistance proteins, and Ca^2+^, MAPK, ROS, and hormonal signaling processes, as well as transcription factors (TFs) associated with defense responses. Furthermore, the levels of anti-fungal metabolites were regulated by MeJA pre-treatment and *A. alternata* infection. To verify the function of these hub genes, inoculation assays with CmWRKY6 transgenic strains showed that CmWRKY6 positively regulated resistance to black spot disease in the chrysanthemum. Our research presents insight into the overall mechanism of JA signaling that drives defense responses against *A. alternata* in chrysanthemum, which will aid the screening of relevant candidate anti-fungal elicitors that could serve as ecologically friendly disease control agents.

## Results

### MeJA-treated chrysanthemum leaves reduced *A. alternata* susceptibility

**Fig. 1:**
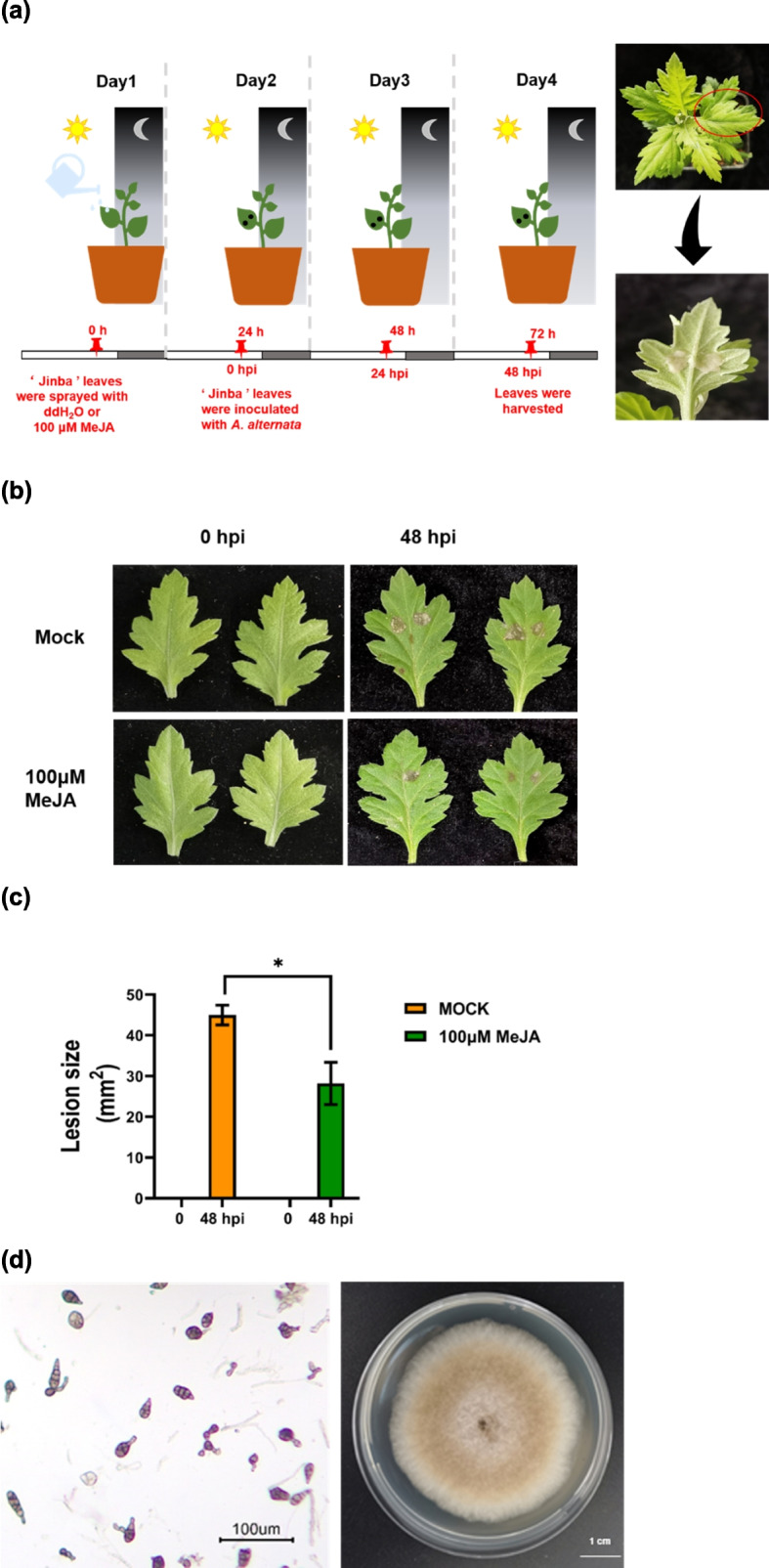
Decreased susceptibility of chrysanthemum leaves to *A. alternata* after pre-treatment with 100 μM MeJA. **a** *C. morifolium* ‘Jinba’ was pre-treated with 100 μM MeJA and inoculated with *A. alternata*. Controls were treated with distilled water. **b** Leaf damage caused by *A. alternata* 48 hpi in control and MeJa-treated leaves. **c** Disease severity was determined by measuring the lesion area (mm^2^) of leaves 48 hpi. Data are presented as the mean ± standard error of four biological replicates. *P ≤ 0.0001 compared to control, as calculated by two-way ANOVA. **d** Pathogens of *A. alternata*. The left part shown that the conidiophores morphology of *A. alternata* at a magnification of × 10. The right part shown that the colony of *A. alternata* growing on potato dextrose agar medium after a week

JAs has been shown to reduce the susceptibility to necrotrophic pathogen infection [32, 33]. To test whether MeJA treatment of chrysanthemum leaves could reduce susceptibility to *A. alternata*, we exogenously sprayed whole *C. morifolium* plants with MeJA before leaves were inoculated with *A. alternata*. After 48 h post-inoculation (hpi), the leaves treated with 100 μM MeJA displayed significantly lower decay in comparison to the control; however, the sensitivity increased with the higher MeJA concentrations (Figs. 1, S1). These results indicated that exogenous 100 μM MeJA induced *A. alternata* resistance in Chrysanthemum.

### MeJA treatment of chrysanthemum caused transcriptional reprogramming following *A. alternata* infection

**Fig. 2:**
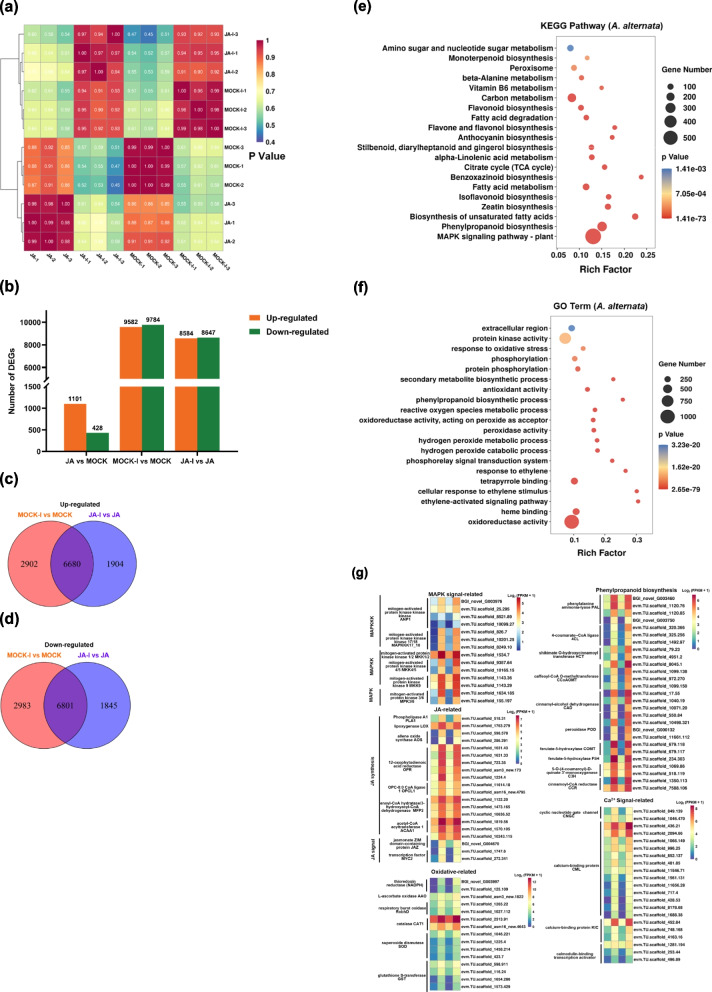
The DEGs, Venn diagrams, and enrichment analysis of chrysanthemum after JA pre-treatment and *A. alternata* infection. **a** Heatmap depicting pairwise Pearson correlation of gene expression values of all samples. **b** Bar graph showing total number of upregulated (orange) and downregulated (green) DEGs in JA vs. MOCK, MOCK-I vs. MOCK, and JA-I vs. JA groups. **c** Venn diagrams presenting the distribution of up-regulated DEGs after JA pre-treatment and *A. alternata* infection. **d** Venn diagrams presenting the distribution of down-regulatedDEGs after JA pre-treatment and *A. alternata* infection. **e** KEGG and (**f**) GO enrichment analysis of the upregulated genes shared between MOCK vs. MOCK-I and JA vs. JA-I groups. **g** Heat map presenting the normalized to Log_2_ (FPKM + 1) of DEGs. Rows are centered based on the average FPKM. From left to right is MOCK, MOCK-I, JA, and JA-I groups

To further explore the function of JA signaling in *A. alternata* defense responses at the transcriptome level, we utilized RNA-seq analysis of *C. morifolium* plants sprayed with 100 µM MeJA or deionized water as a mock with or without *A. alternata* inoculation. Approximately 90% of the reads for each sample were mapped to the reference chrysanthemum genome (https://doi.org/10.6084/m9.figshare.21655364.v2) (Table S1), and the biological replicates for each treatment showed good correlation (Fig. 2a). The differentially expressed genes (DEGs) were identified by comparing JA group versus MOCK group (JA vs. MOCK), MOCK-I group versus MOCK group (MOCK-I vs. MOCK), and JA-I group versus JA group (Fig. 2b). The JA vs. MOCK showed a minimal impact on gene expression, with only 1101 and 428 genes exclusively upregulated and downregulated, respectively. The MOCK-I group showed a significant change in gene expression, with 2902 and 2983 genes exclusively upregulated and downregulated, respectively (Figs. 2c, d), which were self-activated genes independent of JA signaling after inoculation. In contrast, MeJA pre-treatment prevented this change in gene expression post-infection (JA-I vs. JA), with 1904 and 1845 genes upregulated and downregulated, respectively, that were affected by the JA-I treatment. Clearly, pre-treatment with JA reduced infection-induced gene expression, which resulted in a decreased susceptibility to the fungus. The JA-treated leaves are relevant candidates for understanding the mechanism of reduced susceptibility to *A. alternata*. The fact that 6680 DEGs were upregulated both in MOCK-I vs. MOCK and in JA-I vs. JA groups suggested that inoculation induced a response similar to that of the JA-I group and that these genes are pivotal to elucidating JA signaling-mediated defense responses against *A. alternata* (Fig. 2c).

The differentially expressed genes, including those upregulated by MeJA pre-treatment, in MOCK-I, and JA-I groups were classified by gene ontology (GO) analysis and mapped onto metabolic and regulatory pathways using the MAPMAN tool [34]. The MeJA pre-treatment affected the expression of genes associated with metabolic and regulatory pathways and upregulated genes involved in cell wall integrity (cell wall and lipids), terpenes, flavonoids, phenylpropanoids, and phenolic metabolism (Fig. S2a). In JA vs. MOCK group, up-regulated regulatory genes involved in protein modification and degradation, receptor kinases, Ca^2+^ regulation, TFs, and phytohormones (Fig. S2b). Additionally, changes in the expression of secondary metabolic genes involved in anti-oxidative and anti-fungal molecules, including phenylpropanoids, flavonoids and derivatives, glucosinolates, lignin and lignans were observed (Fig. S3a). In mock pre-treatment leaves, MAPMAN-based analysis of the genes that were upregulated following infection revealed a dramatic effect in almost all metabolic and regulatory pathways (Fig. S4a). In contrast, in the MeJA pre-treated group, infection only affected the expression of small number of metabolic genes, including the upregulation of genes involved in cell wall integrity, such as those associated with the biosynthesis of wax, flavonoids, and lipids (Fig. S4c). It is worth noting that almost all regulatory pathways, such as receptor kinases, Ca^2+^ regulation, TFs, and phytohormones, in plants pre-treated with MeJA before infection were positive regulation compared to controls. These results showed that *A. alternata* infection caused significant changes in metabolic pathways, whereas pre-treatment with MeJA prevented this reaction and maintained the expression of defense-related regulatory genes.

We focused on the common up-regulated genes in JA-I vs. JA and MOCK-I vs. MOCK to explore the mechanism of JA signaling-mediated defense responses against *A. alternata*. The 6680 commonly up-regulated genes were classified by the Kyoto Encyclopedia of Genes and Genomes (KEGG) and GO enrichment analysis to assess biological functions (Figs. 2e, f, g, S3b). The results of the analysis showed that MAPK signaling pathway-plant (ko04016, 582 DEGs), phenylpropanoid biosynthesis (ko00940, 275 DEGs), zeatin biosynthesis (ko00908, 102 DEGs), alpha-linolenic acid metabolism (ko00592, 82 DEGs), flavonoid biosynthesis (ko00941, 86 DEGs), anthocyanin biosynthesis (ko00942, 34 DEGs), and peroxisome (ko04146, 91 DEGs) genes were significantly enriched in common up-regulated genes (Fig. 2e). Moreover, GO analysis showed that the DEGs were considerably enriched in oxidative stress processes, including oxidoreductase activity (GO:0016491, 1026 DEGs), hydrogen peroxide catabolic processes (GO:0042744, 95 DEGs), peroxidase activity (GO:0004601, 110 DEGs), ROS metabolic processes (GO:0004601, 96 DEGs), and the ET response pathway, including ethylene-activated signaling pathway (GO:0009873, 63 DEGs), cellular response to ethylene stimulus (GO:0071369, 63 DEGs), and response to ethylene (GO:0009723, 64 DEGs) (Fig. 2f). The MAPMAN-based analysis of the genes showed that common up-regulated genes had a great effect in secondary metabolic pathways, especially phenylpropanoids, phenols, flavonoids and derivatives, lignin and lignans (Fig. S3b). These results indicated that JA could regulate the complex biological pathways of chrysanthemum inoculated with *A. alternata*.

The genes in the major enrichment pathways were primarily involved in MAP kinases, Ca^2+^ signaling, ROS regulation, JA and ET signaling, and phenylpropanoid biosynthesis (Fig. 2g). For instance, regulatory genes encoding the cyclic nucleotide gate channel calcium-binding protein, ROS scavenging enzyme-like L-ascorbate oxidase (AAO), catalase (CAT1), superoxide dismutase (SOD), peroxidase (POD), and glutathione S-transferase (GST); as well as genes encoding key enzymes of phenylpropanoid biosynthesis such as phenylalanine ammonia-lyase (PAL), 4-coumarate-CoA ligase (4CL), shikimate O-hydroxycinnamoyl transferase, caffeoyl-CoA O-methyltransferase, cinnamyl-alcohol dehydrogenase, ferulate-5-hydroxylase, ferulate-5-hydroxylase, 5-O-(4-coumaroyl)-D-quinate 3'-monooxygenase, cinnamoyl-CoA reductase and POD; genes encoding key enzymes of the JA synthesis pathway, such as phospholipase A1, lipoxygenase, allene oxide synthase, 12-oxophytodienoic acid reductase, OPC-8:0 CoA ligase 1, enoyl-CoA hydratase/3-hydroxyacyl-CoA dehydrogenase, and acetyl-CoA acyltransferase 1. Among the common genes, 23 genes that were associated with the defense response showed a higher induction in JA-I vs. JA than that in MOCK-I vs. MOCK, including pathogenesis-related proteins (PDF1.2, PR10, PR1, RPS4, and RPS2), proteins associated with strengthening of the cell wall barrier (GT61, CESA, and ChiB), and defense-related molecular chaperones (HSP90) (Fig. S3; Table S3). This analysis suggested that these genes might contribute to resistance to *A. alternata*.

**Fig. 3:**
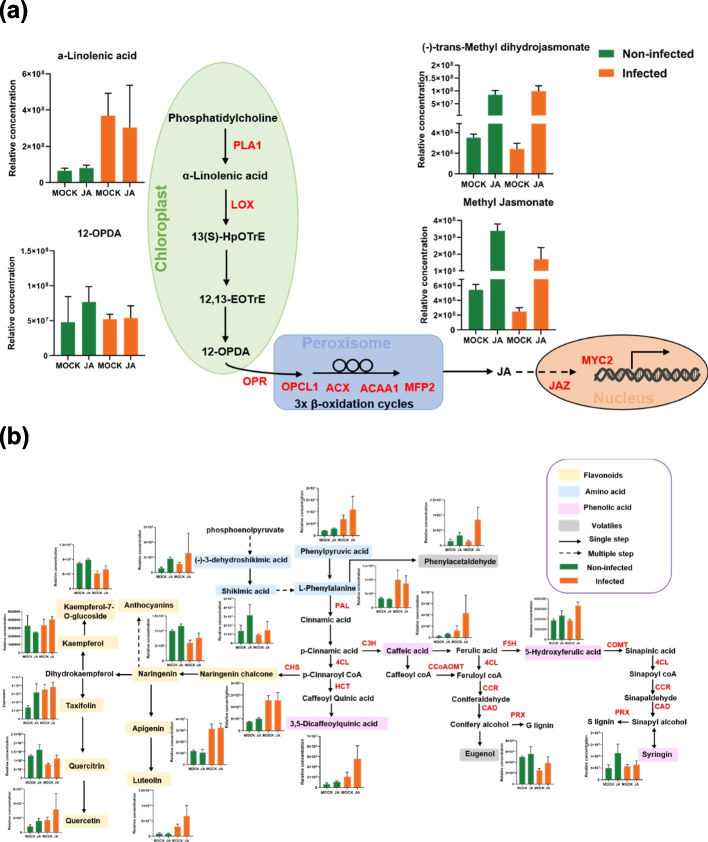
Metabolic changes in chrysanthemum leaves after JA-treatment and *A. alternata* infection. **a** Changes in JA synthesis-related metabolites levels in chrysanthemum leaves in MOCK, MOCK-I, JA, JA-I groups. **b** Changes in phenolic acid levels in chrysanthemum leaves in MOCK, MOCK-I, JA, JA-I groups. Changes in the levels of metabolites were analyzed by LC–MS/MS. Values represent means from three biological replications ± standard error. Green bars indicate metabolite levels in non-infected leaves and orange bars represent metabolite levels after infection. MOCK, control; JA, MeJA pre-treated. Note: the KEGG pathways were retrieved from the Kanehisa Laboratories [35]

The metabolomics results also verified that downstream metabolic changes were involved in JA biosynthesis, lignin biosynthesis, and oxidative stress processes. Exogenous MeJA treatment in plant leaves led to the accumulation of numerous derived phenolics, phenylpropanoids, and flavonoids (Fig. 3). In this study, MeJA pre-treatment caused a significant increase in endogenous JA levels such as methyl jasmonate (61.1-fold) and (˗)-trans-methyl dihydrojasmonate (23.24-fold), and increased downstream metabolites included shikimic acid (1.29-fold), phenylacetaldehyde (1.27-fold), caffeic acid (1.51-fold), 3,5-dicaffeoylquinic acid (0.72-fold), syringin (1.27-fold), quercetin (0.94-fold), taxifolin (1.28-fold), and cyanidin (0.75-fold) when compared with the controls. These findings suggested that MeJA pre-treating the leaves led to an absorption of JA and subsequent activation of downstream metabolites. The primary impact of MeJA pre-treatment was noticed after fungal infection. In mock-treated leaves, fungal infection led to a substantial reduction in the phenylalanine derived volatile eugenol (reduced by 50%) and flavonoid derived naringenin (reduced by 40%), pethidine (reduced by 55%), and quercitrin (reduced by 39%). However, luteolin (2.65-fold), 3,5-dicaffeoylquinic acid (2.3-fold), taxifolin (1.55-fold), and naringenin chalcone (2.47-fold) increased in the infected leaves of non-treated leaves. Interestingly, MeJA pre-treatment increased or further promoted the accumulation of these metabolites (Figs. 3b and S5), suggesting that fungal infection can negatively affect the antioxidant system of plants, whereas MeJA pre-treatment prevented the decline in these metabolites and maintained intracellular ROS homeostasis. In MeJA-treated leaves, the metabolic impacts following infection included an accumulation of flavonoid-derived peonidin (66.46-fold), pethidine (6.24-fold), and monolignols 4,5-dicaffeoylquinic acid (5.06-fold) and 5-hydroxyferulic acid (0.42-fold) (Figs. 3b, S5).

### Identification of TFs involved in JA treatment and *A. alternata* infection

**Fig. 4:**
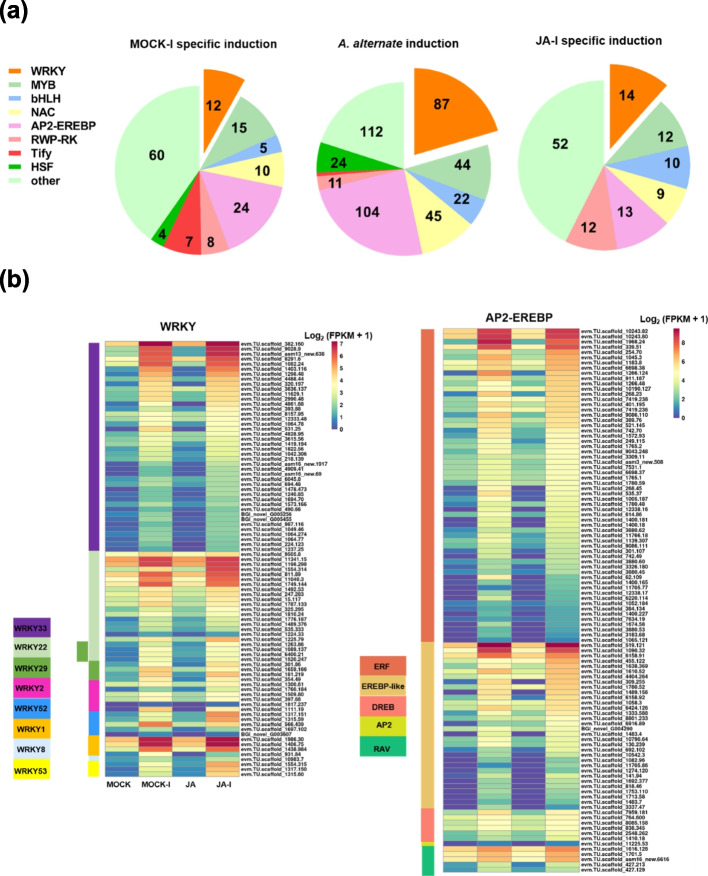
Differentially expressed transcription factors in response to JA-treatment and *A. alternata* infection. **a** Classification of transcription factors. **b** Heat map of the normalized Log_2_ (FPKM + 1) of WRKY and AP2-EREBP transcription factors in the common upregulated DEGs. From left to right is MOCK, MOCK-I, JA, and JA-I. MOCK, control; MOCK-I, control infected group; JA-I, MeJA pre-treated and infected group; JA, MeJA-pre-treated group

TFs form the core of the gene regulatory network, mediating transcriptional reprogramming in the reaction to phytopathogens. It has been demonstrated that members of the TF family, such as WRKY, AP2/EREBP, NAC, and MYB, contribute to defense against *A. alternata* [36–38]. In our study, a large number of TFs were identified through DEG analysis that were specifically or commonly upregulated. Therefore, the host defense response could be significantly impacted by variations in TF expression. These differentially expressed TFs included WRKY, AP2/EREBP, MYB, and NAC (Fig. 4a). Of the 6680 common genes, 425, including 87 WRKYs and 104 AP2/EREBPs, encoded TFs (Fig. 4a). Most of these WRKYs belonged to WRKY33 and WRKY22 families. The AP2/EREBPs belonged to the EREBP subfamily, such as ethylene response factor (ERF), dehydration response element binding protein (DREB), and other proteins (EREBP-like; Fig. 4b). We identified 122 TFs specifically in the JA-I group, including 14 WRKYs and 13 AP2/EREBPs (Figs. 4a, S8). One forty five genes of the DEGs exclusively found in MOCK-I were classified as TFs. MYB (15 members) and AP2/EREBP (24 members) were the two TFs with the most annotations.

### Validation of differential gene expression using reverse transcription quantitative PCR (RT-qPCR)

**Fig. 5:**
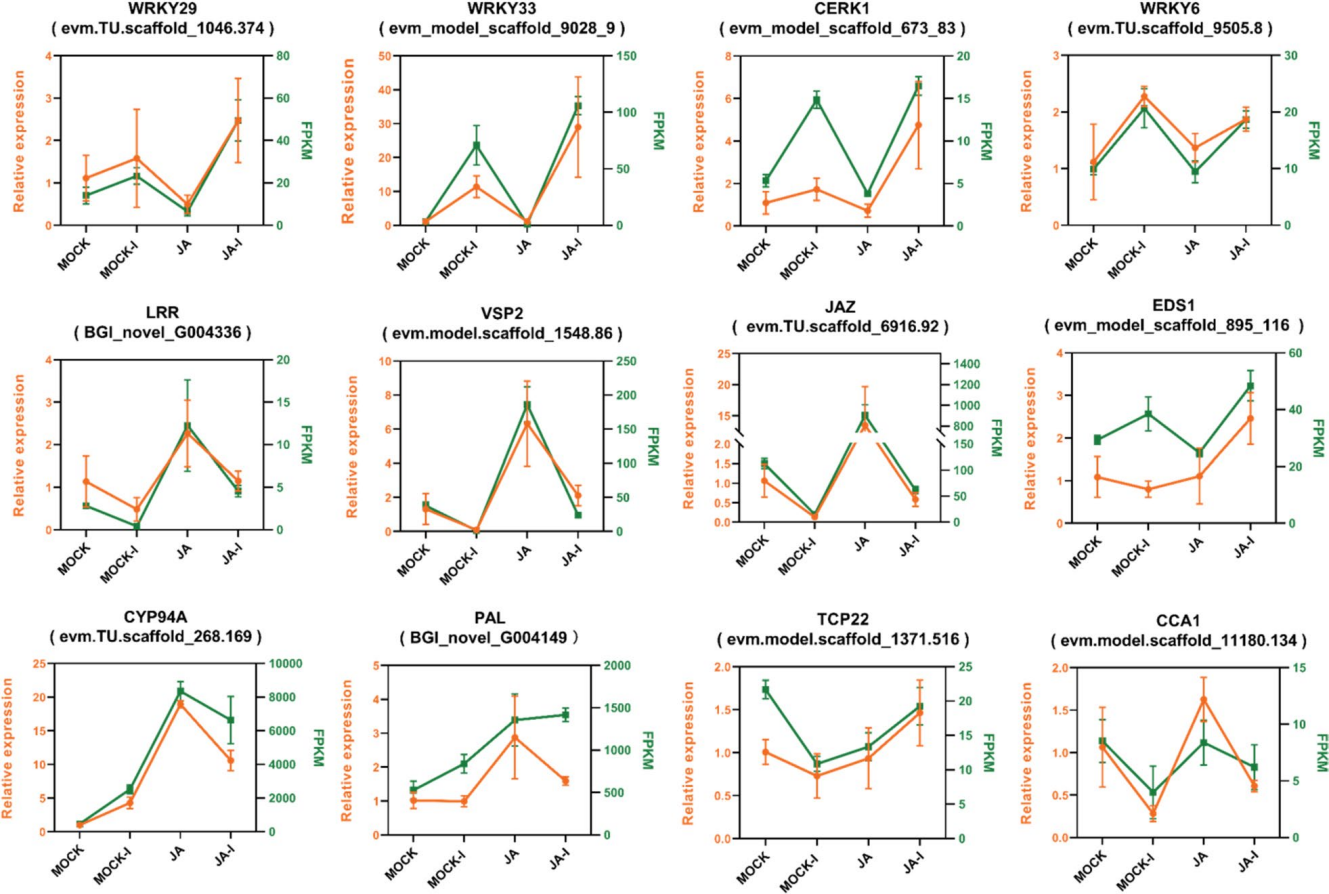
Changes in gene expression levels after JA pre-treatment and *A. alternata* infection. The left vertical axis represents relative gene expression level from RT-qPCR (orange) and the right vertical axis represents FPKM from RNA-seq (green). MOCK, control; MOCK-I, control infected group; JA-I, MeJA pre-treated and infected group; JA, MeJA-pre-treated group

To validate the RNA-seq results, 12 genes were randomly selected from a total 6680 common genes for RT-qPCR. The expression of WRKY29 (evm.TU.scaffold_1046.374), WRKY33 (evm_model_scaffold_9028_9), WRKY6 (evm.TU.scaffold_9505.8), and CERK1 (evm_model_scaffold_673_83) was induced by *A. alternata* infection. The expression of LRR receptor-like kinase (BGI_novel_G004336), VSP2 (evm.model.scaffold_1548.86), JAZ (evm.TU.scaffold_6916.92), ESD1 (evm_model_scaffold_895_116), CYP94A (evm.TU.scaffold_268.169), PAL (BGI_novel_G004149), and CCA1 (evm.model.scaffold_11180.134) was induced by MeJA-treatment. Similar upregulation or downregulation expression patterns were seen in the qRT-PCR and RNA-seq data (Fig. 5), indicating that our transcriptome data was reliable.

### Overexpression of *CmWRKY6* confers chrysanthemum resistance to black spot disease

**Fig. 6:**
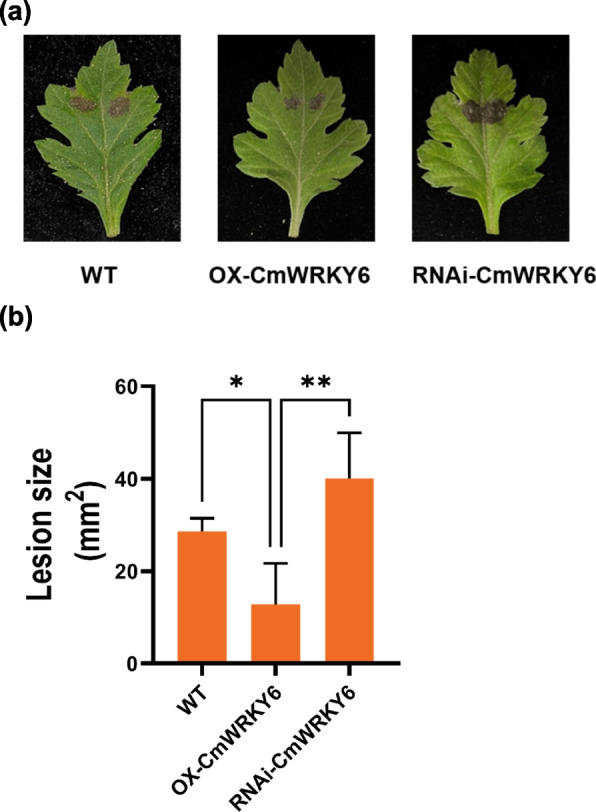
Overexpression of *CmWRKY6* confers resistance to black spot disease in chrysanthemum ‘Jinba’. **a** The *A. alternata* infection phenotypes in inoculated leaves of WT, OX-CmWRKY6 and RNAi-CmWRKY6 plants, respectively. **b** Disease severity was determined by measuring the lesion area (mm^2^) of leaves 48 hpi. Data are presented as the mean of four replicates ± standard error. Asterisks (*) depict statistically significant differences for each time interval between the different treatments, as calculated by a two-way ANOVA (*P ≤ 0.05, ** P ≤ 0.01)

To further verify the reliability of the results, we choose WRKY6 (evm.TU.scaffold_9505.8), which was induced by *A. alternata*, to generate overexpressed and silenced (RNA interference [RNAi]) CmWRKY6 chrysanthemum plants [39]. Inoculation assays demonstrated that compared with wild-type (WT), the CmWRKY6 overexpressing (OX-CmWRKY6) lines had enhanced resistance to black spot disease, with a lesion area that was reduced by 55%. Conversely, the CmWRKY6 silenced lines (RNAi-CmWRKY6) displayed enhanced susceptibility, with a lesion area that increased by 40% (Fig. 6). These results further validated the upregulated genes that were identified by comparing MOCK-I vs. MOCK with JA-I vs. JA groups, indicating that they are relevant candidates for understanding the mechanism of JA-induced *A. alternata* resistance.

## Discussion

### JA enhances resistance to fungal pathogen in plants

To respond to fungal attacks, plants produce defense-related compounds and different phytohormones. High-throughput data obtained through liquid chromatography tandem mass spectrometry (LC–MS/MS) and RNA-seq technology can objectively and comprehensively reflect the global metabolic change and transcriptional expression associated with pathogen responses [40], such as the resistance induced by different elicitors or natural molecules. At present, many studies have shown that exogenously applied elicitors or natural molecules can induce plant defense responses. For instance, high phenylalanine concentrations reduce the susceptibility to *Botrytis cinerea* in petunia, Arabidopsis, tomato leaves, and chrysanthemum flowers [41, 42]. Exogenous MeJA also induced resistance to fungal pathogen in potato [14] and rose leaves [15]. Here, we showed that MeJA pre-treatment decreased the susceptibility of chrysanthemum leaves to *A. alternata* (Fig. 1), indicating that treatment with elicitors or natural molecules is an effective mode of enhancing pathogen resistance in a range of plant species. However, the mechanisms connecting *A. alternata* infection with JA signaling are not completely clear, especially in chrysanthemum plants. Therefore, we monitored the dynamics of metabolites and transcriptomes in leaves after MeJA pre-treatment and *A. alternata* infection to explore JA-dependent cross-talk, signaling, and defense responses in disease-resistance systems. These results further deepen our understanding of the JA-mediated mechanisms underlying resistance to *A. alternata* infection in chrysanthemum.

### JA enhances the secondary metabolism in chrysanthemum after *A. alternata* infection

Similar to a previous study, MeJA pre-treatment influenced the expression levels of JA synthesis genes and induced the accumulation of endogenous JA such as methyl jasmonate and (˗)-trans-methyl dihydrojasmonate (Figs. 3a, S2b). Inducing the expression of genes associated with secondary metabolism (Fig. S3a) further increased the concentrations of secondary metabolites [14, 15], specifically phenylpropanoids and flavonoids, including shikimic acid, caffeic acid, 3,5-dicaffeoylquinic acid, syringin, quercetin, and taxifolin (Fig. 3b), which have anti-fungal and anti-oxidative activity [42]. The MeJA pre-treatment prevented the reduction in the levels of the volatiles, such as phenylacetaldehyde and eugenol in the JA-I group (Fig. 3b). This could be as a result of the enhanced carbon flow towards the formation of eugenol in MeJA-pretreated leaves following infection, consistent with the release of volatiles being associated with pathogen resistance [43]. Additionally, MeJA pre-treatment resulted in transcriptional reprogramming of a group of genes involved in the cell wall, lipids, Ca^2+^ signaling processes, and hormonal signaling processes, which are connected to plant defense responses to pathogen attack (Fig. S2). These results confirmed the JA can regulate specific primary and secondary metabolic processes [14, 15].

The influence of MeJA pre-treatment, both at the transcriptomic and metabolic levels suggested that the JA priming of plant leaves enabled a rapid immune response after *A. alternata* infection through the activation of a diverse set of defense mechanisms. In this study, plants that received the MeJA pre-treatment showed small transcriptional changes after *A. alternata* infection in contrast to the drastic increase in mock-treated leaves after infection (Figs. 2b, c). This difference may be related to the overall early pathogen defense response induced by exogenously applied JA [14]. To further explore the mechanism of JA-induced resistance against *A. alternata*, we focused on the 6680 genes upregulated in both MOCK-I vs. MOCK and in JA-I vs. JA groups. The common DEGs played a central role in coordinating different pathways, and accounted for 78% of the DEGs in JA-I and 70% of the DEGs in MOCK-I (Fig. 2c), reflecting the relationship between JA signaling and *A. alternata*. The RNA-seq analysis showed that the commonly upregulated DEGs were involved in MAPK signaling pathway, secondary metabolism (flavonoid, anthocyanin, and phenylpropanoid biosynthesis) and JA biosynthesis (α-linoleic acid metabolism). The GO term enrichment analysis highlighted that the DEGs were mainly associated with oxidative stress processes (Figs. 2d, e). These findings indicated that various metabolic production and defense pathways are possibly induced through the accumulation of secondary metabolites and ROS scavenging in chrysanthemum.

### JA enhanced the cell wall integrity maintenance system

The production of ROS often initiates various defense responses that help plants in fighting off pathogen attacks, which can include enhancing the cell wall barrier and regulating Ca^2+^ signaling, as ROS can function as Ca^2+^ sensors [44]. We found the upregulation of Ca^2+^ signaling and ROS metabolic genes in the JA and JA-I groups (Fig. 2g), suggesting the interplay between ROS and Ca^2+^ signals contributed to the defensive response. The overexpression of ROS can promote cell injury; therefore, maintaining intracellular ROS homeostasis can promote plant defense response [45]. Previous reports have provided evidence that JA prevents excess ROS generation [46] and promotes accumulation of antioxidant enzymes [47]. Our findings show that the DEGs that encoded antioxidant enzymes (two CAT1s, four SODs, one AAO, four GSTs, and four PODs) were significantly upregulated in the JA-I group (Fig. 2g). Furthermore, the levels of anti-oxidative metabolites, including flavonoids (luteolin and quercetin), phenolic acids (3,5-dicaffeoylquinic acid), and anthocyanins (cyanidin and pethidine), were increased (Figs. 3b, S5b).

ROS and Ca^2+^ signaling also mediate defense mechanisms through cell wall strengthening and re-organization [48], and the induced expression of genes related to cell wall biosynthesis and wax release may also act as signals for cell wall remodeling to defend against pathogen infection [49]. Different classes of flavonoids and lignin are synthesized via the phenylpropanoid pathway [50]. Lignin is a structural component of the cell wall and the expression of its biosynthetic pathway genes enhance plant defense [51–53]. The expression of genes encoding key enzymes of the phenylpropanoid pathway was significantly upregulated following *A. alternata* infection in chrysanthemum (Fig. 2g). Notably, one PAL (BGI_novel_G003750), one 4CL (evm.TU.scaffold_320.366), and three POD (evm.TU.scaffold_550.84, BGI_novel_G000132, evm.TU.scaffold_11661.112) genes were upregulated in JA-I vs. JA compared with that in MOCK-I vs. MOCK (Table S2). The downstream lignin synthesis substrate monolignols also accumulated in the MeJA-treated plants (Fig. S5a) after enzymatic oxidization and the subsequent radical coupling in the cell walls and formed a heterogenous polymer that constitutes lignin [54], suggesting that this might contribute to the stronger physical barrier for plant defense.

### JA enhanced pathogen‐induced MAPKs signaling

MAPK cascades participate in many signal‐transferring processes, are essential signaling modules downstream of receptors or sensors that detect endogenous stimuli like PAMPs and effectors, and play crucial roles in signal transduction in response to phytohormones and environmental challenges [55–58]. MAPKs further transmit and amplify these signals through the stepwise phosphorylation of mitogen-activated protein kinase kinases (MAPKKs) and mitogen-activated protein kinase kinases (MAPKKKs). The *A. alternata* infection in chrysanthemum caused the induction of MAPKs, MAPKKs, and MAPKKKs signaling events (Fig. 2g). In Arabidopsis, MPK3/MPK6 and their orthologs were proposed to share a subset of defense responses [59, 60] that affect many downstream transduction pathways. For example, MPK3/MPK6 cascade and Ca^2+^ signaling pathway crosstalk regulate the biosynthesis of camalexin [61, 62]. MPK3/MPK6 regulate ET biosynthesis during pathogen attack [63, 64]. Additionally, the functions of the MKK4/MKK5–MPK3/MPK6 module in plant immunity have been identified [65], and the search for the MAPKKK(s) upstream of the MKK4/MKK5–MPK3/MPK6 module has progressed in recent years [66, 67], but there is still a gap in our understanding of its mechanism. In this study, MPK3 (evm.TU.scaffold_1634.185 and evm.TU.scaffold_155.197), MKK4/5 (evm.TU.scaffold_9357.64 and evm.TU.scaffold_10165.15), and ANP1 (evm.TU.scaffold_10099.27 and evm.TU.scaffold_826.7) were up-regulated by *A. alternata* and were more significantly up-regulated in the JA-I group (Fig. 2g; Table S2). This indicated that JA signaling may regulate Ca^2+^ signaling and the synthesis of phytoalexin or activate ET signaling by upregulating the expression of MPK3 to produce disease resistance. Conversely, it suggests that JA signaling might occur via an ANP1-MKK4/5–MPK3 cascade to activate immune signaling, which could be related to oxidative signal transduction. Early studies have shown that H_2_O_2_ can activate the specific Arabidopsis MAPKKK, ANP1, to initiate a phosphorylation cascade involving the stress MAPKs, AtMPK3 and AtMPK6 [68]. Recently, MKK4/MKK5 has been reported to regulate plant defense pathways, including ROS production and the synthesis of ET and SA [69], suggesting that ANP1-MKK4/MKK5–MPK3/MPK6 is an oxidative stress-activated mitogen-activated protein kinase cascade in plants. Conclusively, JA signaling is one of the main pathways of JA-induced resistance, which can further amplify ROS regulation and ET signaling through the ANP1-MKK4/MKK5–MPK3 cascade to regulate immune responses.

### The role of transcription factors in the JA signaling-induced defense response

TFs play key roles in coordinating large-scale transcriptional reprogramming that resolve plant immune mechanisms [70, 71]. Numerous TFs that act as essential players in JA signal transduction have been discovered using forward and reverse genetic methods [72]. In our study, scanning through the shared DEGs between the JA-I and MOCK-I groups, we identified 104 AP2-EREBP and 87 WRKY TFs, which accounted for the two largest proportions of overlapping TFs (Fig. 4), suggesting that AP2-EREBP and WRKY family members play direct roles in JA-triggered immunity. At present, studies have indicated that AP2-EREBP TFs regulate the signal transduction pathways of numerous phytohormones, including ET, ABA, cytokinin (CTK), and JA [73–75], activating inducible defense responses in plants. The AP2-EREBP family is divided into five subfamilies: AP2, ERF, DREB, ABI3/VP1 (RAV)-related, and other EREBP-like [76]. In the present study, most of the AP2-EREBP TFs induced by JA and *A. alternata* infection belonged to ERF (60 members, 57.7%, Fig. 4b). Previous studies reported that JA and ET synergistically activate defense signaling against necrotrophic pathogens [77, 78]. Moreover, WRKY TFs participate in phytohormone-mediated signaling pathways and transcriptional reprogramming associated with plant defense responses like the MAPK signaling cascade [79, 80]. NaWRKY3 and NaWRKY6 can regulate the synthesis of JA to mediate pathogen defense responses [81]. WRKY33 positively regulates target genes involved in the biosynthesis of the antimicrobial compound camalexin and JA/ET downstream signaling [82, 83], and functions as a key transcriptional regulator required for immunity in Arabidopsis towards *Botrytis cinerea* [84, 85]. In this study, most of the WRKY TFs that regulate immune responses induced by JA and *A. alternata* belonged to WRKY33 (42 members, 48%), followed by WRKY22 (22 members, 25%), and WRKY29 (8 members, 14%) (Fig. 4b). The WRKY33 and WRKY22/29 TFs were significantly upregulated in JA-I compared with those in the MOCK-I group (Fig. S7). This is potentially the reason that MeJA pre-treatment had a stronger induction of resistance genes than the MOCK-I group (Fig. S6, Table S2). Three TFs were annotated as WRKY53 (evm.TU.scaffold_1554.315, evm.TU.scaffold_1317.150 and evm.TU.scaffold_1315.60) in chrysanthemum, which were more significantly up-regulated in JA-I group (Figs. 4b, S7), suggesting that WRKY53 may be mediated by JA signaling in response to pathogenic fungal infection. Additionally, 13 AP2-EREBP and 14 WRKY TFs were detected specifically in JA-I, which may play important positive regulatory roles in mediating the JA signal pathway against *A. alternata* in chrysanthemum. Our recent research shows that CmWRKY6 negatively regulates the resistance to *Fusarium oxysporum* [39], and in the present study, WRKY6 was upregulated in both JA-I vs. JA and MOCK-I vs. MOCK groups, and the CmWRKY6 overexpression line had reduced susceptibility to black spot disease compared to the control. Differences in pathogenicity between these reports might be because *F. oxysporum* is a soilborne plant pathogen whose hypha penetrates plant roots rather than invading leaves like *A. alternata*.

## Conclusions

**Fig. 7:**
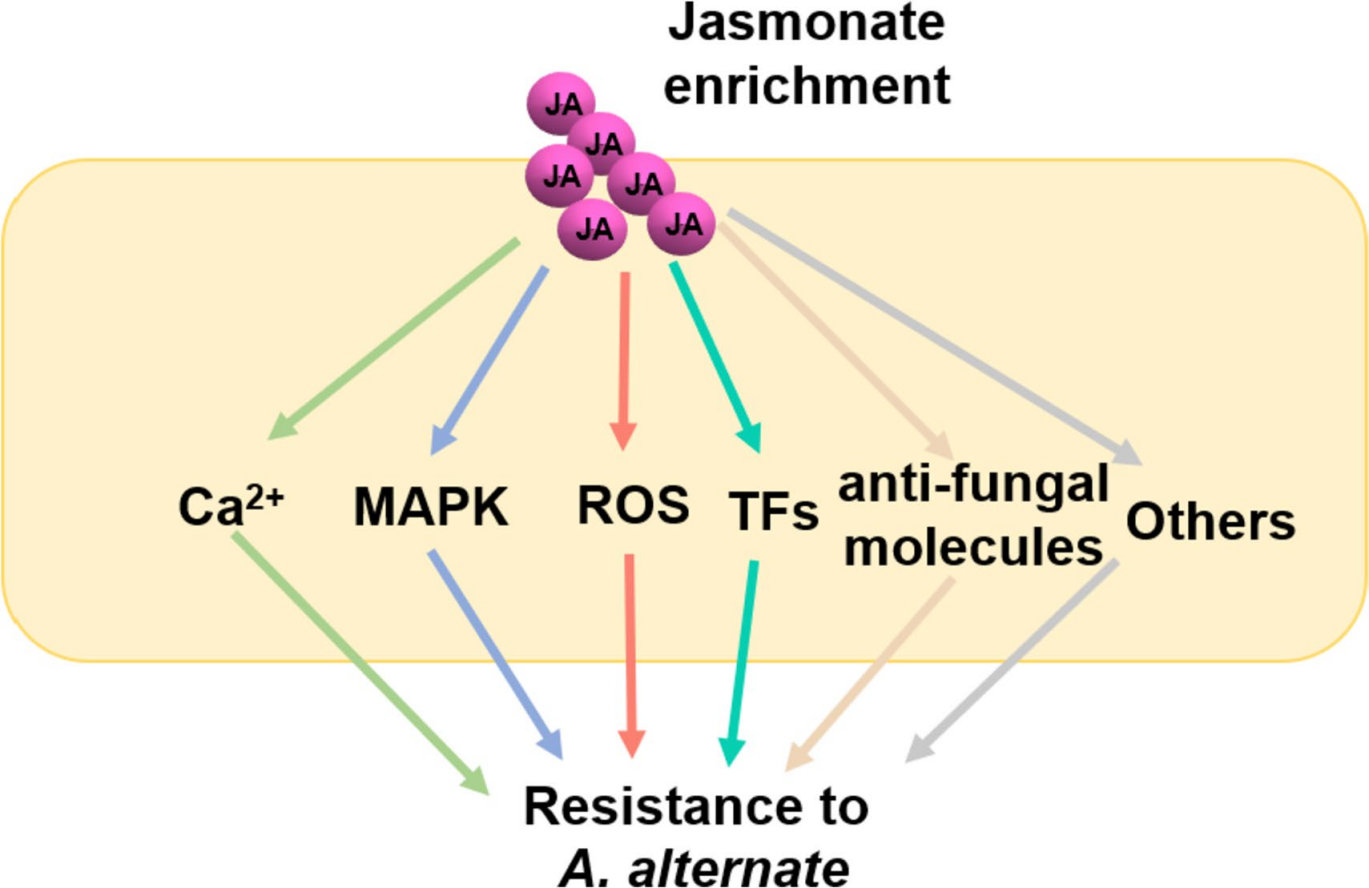
Hypothetical model of the jasmonate-induced defense mechanism. JA signaling activates endogenous Ca^2+^, ROS, MAPK, and TF signaling transduction and the accumulation of anti-fungal molecules, which enhances resistance to *A. alternate*

We have presented experimental evidence that MeJA pre-treatment is an effective strategy to control black spot in plants. JA enrichment promotes multilayered defense responses in plant tissues and improves host immunity by mediating the expression of receptor kinases, TFs, and proteins involved in Ca^2+^ signaling, hormone signaling, cell wall and lipid metabolism pathways, in coordination with the production of anti-fungal and anti-oxidant metabolites. Our results suggest that MeJA pre-treatment mediates the transcriptional reprogramming of defense response activation before fungal infection. During pathogen attack, MeJA pre-treatment probably activates the ANP1-MKK4/MKK5–MPK3 cascade, ROS and ET signaling events, and induces TFs that mediate gene regulatory networks in response to *A. alternata*, along with promoting the production of anti-fungal and anti-oxidant metabolites. Therefore, the role of JA signaling in positively regulating plant immunity is dependent on crosstalk among multiple signaling pathways (Fig. 7). The findings of the present study identified promising candidates with anti-fungal and anti-oxidant characteristics that may serve as ecologically friendly pathogen control agents.

## Materials and methods

### Plant material and *A. alternata* culture

The chrysanthemum cultivar ‘Jinba’ was provided by the Chrysanthemum Germplasm Resource Preserving Center at Nanjing Agricultural University (Nanjing, China). Rooting cuttings of similar growth stage were transplanted in a 3:1 mixture of vermiculite and perlite without fertilizer. Chrysanthemums were cultivated in an illumination incubator with a photoperiod of 16 h light/8 h dark at 28 °C and 70% humidity. After the transplants had up to 10 mature leaves, they were used in the experiments.

The *A. alternata* strain used for this study was isolated and identified from typical infected leaves of Chrysanthemum ‘Fubaiju’ at our laboratory. The test strain was moved to potato dextrose agar solid medium, where it was grown for around a week at 28 °C. Thereafter, fungal cakes were transferred into 200 mL potato dextrose water liquid medium and grown overnight at 28 °C with shaking at 180 rpm before inoculation assays were conducted.

### Inoculation with *A. alternata* and MeJA treatments

*C. morifolium* leaves were sprayed with distilled water or 100 µM MeJA (Sigma-Aldrich, Darmstadt, Germany) until all leaves of each plant were wet. Twenty-four hours after exogenous elicitation, two leaves on each plant were inoculated with *A. alternata* (Fig. 1a). Each inoculation site was about 1 cm in diameter. There were four treatments: 100 µM MeJA pre-treatment (JA group), 100 µM MeJA pre-treatment and inoculation (JA-I group), mock (control, MOCK group), mock and inoculation (MOCK-I group). This procedure ensured that each spot was inoculated with a quantitative amount of mycelium. Each groups were cultivated in a controlled environment with a photoperiod of 16 h light/8 h dark at 28 °C and 70% humidity. The lesion area was observed at 48 hpi.

### RNA extraction and RNA-seq library construction

Total RNA was isolated from leaves from the MOCK, MOCK-I, JA, and JA-I groups (three samples per group) at 48 hpi using the RNA extraction kit (Huayueyang Biotechnology, Beijing, China) following the manufacturer’s protocol. All 12 libraries were constructed and sequenced using the using an DNBSEQ platform at BGI (Shenzhen, China) to generate sequence reads.

### Analysis of RNA-seq datasets

To create clean read data, the original raw data was filtered, adapter sequences and poly-N and poor-quality reads were eliminated. After filtering, the clean data were mapped by HISAT (v2.1.0) [86] to the chrysanthemum genome [87], matched to reference gene sequences by Bowtie2 [88], and the gene expression level of each sample was determined using RSEM [89]. Differential expression analysis was performed with DESeq2 [90], using the negative binomial distribution model, the hypothesis test probability (*P* value) was calculated to identify differences in the gene expression data [91]. Genes with fold change ≥ 2, and adjusted *P* value (Q value) ≤ 0.001 were designated as DEGs. Based on the GO (http://geneontology.org) and KEGG (http://www.genome.jp/kegg) notes genes and classifications, the DEGs were functionally classified, and the phyper (https://en.wikipedia.org/wiki/Hypergeometric_distribution) in the R software was applied for KEGG enrichment analysis, whereas the TermFinder package was utilized for GO enrichment analysis (https://metacpan.org/pod/GO::TermFinder). The cutoff point for defining candidate genes as significantly enriched was set at Q value ≤ 0.05.

#### Extraction and LC–MS/MS profiling of metabolites

Leaves from three biological replicates of the MOCK, MOCK-I, JA, and JA-I groups were clipped to eliminate the lesions and immediately stored at liquid nitrogen. The freeze-dried leaves were crushed into a fine powder and used for metabolite extraction. The extracts were used for a further LC–MS/MS analysis after absorbing and filtering. Raw LC–MS/MS data were collected for peak extraction and identification to obtain peak area and identify metabolites, respectively. Data preprocessing was performed using metaX [92]to obtain and identify the isolated metabolic compounds. The identified metabolites were categorized and functionally annotated using KEGG ID, HMDB ID, category, and KEGG pathway in the KEGG and HMDB databases.

#### Validation of RNA-seq data by (RT-qPCR)

Twelve DEGs were chosen at random for RT-qPCR. Primers were designed using PRIMER 5 software (Table S3). RT-qPCR was carried out using an Eppendorf Mastercycler Ep RealPlex 2S fluorescence quantifier (Hamburg, Germany). The manufacturer's instructions were followed while using the 2 SYBR Green qPCR master mix (Bimake) in the reactions. A total of 10.0 μL of SYBR® Premix Ex TaqTM II, 1.0 μL of each 10 μM forward and reverse primer, and 2.0 μL of cDNA template were used in each reaction. The following described the reaction conditions: 95 °C for 10 min, followed by 40 cycles of 95 °C for 15 s, 60 °C for 15 s, and 72 °C for 20 s. CmEF1α (GenBank: AB548817.1) was used as a reference gene, and the 2^−ΔΔCT^ method was used to calculate each gene's relative expression level [93].

### Supplementary Information

**Additional file 1: Fig. S1.** Decreased *A. alternate *susceptibility in chrysanthemum leaves pre-treated with MeJA. (a) *Chrysanthemum morifolium* ‘Jinba’ was pre-treated with 50, 100, and 200 μM MeJA and then inoculated with *A. alternate* before sampling after 48 hpi. Controls were treated with distilled water. (b) Disease severity is expressed as lesion area (mm^2^) of leaves after 48 hpi. Data are presented as the mean of four replicates ± standard error. Asterisks (*) indicate statistically significant differences evaluated for each time interval between the different treatments as calculated by two-way ANOVA (*P ≤ 0.05, ** *P* ≤ 0.01).

**Additional file 2: Fig. S2.** Visualization of gene expression after treatment with JA. (a) Differentially expressed genes related to metabolic pathways. (b) Differentially expressed regulatory genes. The log_2_ (fold change) values of genes significantly upregulated in JA pre-treated leaves compared to controls (MOCK) are presented as red colored squares. Not all differentially expressed genes are presented in the map, only the genes related to metabolic pathways and regulation are displayed. CHO, carbohydrates; OPP, oxidative pentose phosphate pathway; TCA, tricarboxylic acid cycle.

**Additional file 3: Fig. S3.** Visualization of the changes in the secondary metabolic gene expression following JA treatment and *A. alternate* infection. (a) Significant upregulation of secondary metabolic genes in the JA-treated group compared to MOCK. (b) Significant upregulation of secondary metabolic genes in the JA-I group compared to JA-treated group. The log_2_ (fold change) values of differentially expressed genes are represented by red squares.

**Additional file 4: Fig. S4.** Visualization of the changes in metabolic and regulatory gene expression following* A. alternate* infection. (a) Exclusively upregulated metabolic genes in MOCK-I vs. MOCK. (b) Exclusively upregulated regulatory genes in MOCK-I vs. MOCK. (c) Exclusively downregulated metabolic genes in JA-I vs. JA. (d) Exclusively upregulated regulatory genes in JA-I vs. JA. The log_2_ (fold change) values of differentially expressed genes are represented by red squares. Not all differentially expressed genes are presented in the map, only the genes related to metabolic pathways and regulation are displayed. CHO, carbohydrates; OPP, oxidative pentose phosphate pathway; and TCA, tricarboxylic acid cycle.

**Additional file 5: Fig. S5.** Changes in secondary metabolite levels in chrysanthemum leaves after JA treatment and *A. alternate* infection. (a) Changes in monolignol levels in Chrysanthemum leaves in MOCK, MOCK-I, JA, JA-I groups. (b) Changes in anthocyanin levels in Chrysanthemum leaves in MOCK, MOCK-I, JA, JA-I groups. Changes in the levels of metabolites were analyzed by GC/LC-MS. Values represent the means of three biological replicates ± standard error. Green bars indicate metabolite levels in non-infected leaves, while orange bars are the metabolite levels after infection. MOCK, control; MOCK-I, control infected group; JA-I, MeJA pre-treated and infected group; JA, MeJA-pre-treated group.

**Additional file 6: Fig. S6.** Changes in shared defense-related gene expression levels in response to JA treatment and *A. alternate* infection. Heat map presenting normalized Log_2_ (FPKM+1) expression of genes that are more up-regulated in JA-I vs. JA compared to MOCK-I vs. MOCK. Rows are centered based on the average FPKM. MOCK, control; MOCK-I, control infected group; JA-I, MeJA pre-treated and infected group; JA, MeJA-pre-treated group.

**Additional file 7: Fig S7.** Changes in shared TF expression levels due to JA treatment and *A. alternate* infection. Heat map presenting normalized Log_2_ fold change of WRKY TFs that are more up-regulated in JA-I vs JA when compared to MOCK-I vs. MOCK groups. MOCK-I, log_2_ (MOCK-I/MOCK) and JA-I, log_2_ (JA-I/JA).

**Additional file 8: Fig. S8.** Changes in exclusively upregulated TF expression in JA-I vs. JA groups. Heat map of normalized Log_2_ (FPKM+ 1) of exclusively WRKY and AP2-EREBP TFs that are up-regulated in JA-I vs. JA groups. Rows are centered based on the average FPKM. From left to right is MOCK, MOCK-I, JA, JA-I. MOCK, control; MOCK-I, control infected group; JA-I, MeJA pre-treated and infected group; JA, MeJA-pre-treated group.

**Additional file 9: Table S1.** Summary of reads mapping

**Additional file 10: Table S2.** Primer sequences used in qRT-PCR for the validation of dual RNA-seq data.

**Additional file 11: Table S3.** Shortlisted genes from differential expression analysis that have a potential role in defense response to *A. alternate* infection

## Data Availability

The datasets generated during the current study were submitted to the NCBI repository, bioproject PRJNA982184. Chrysanthemum genome used in the study is from the website https://doi.org/10.6084/m9.figshare.21655364.v2.
