# Supplementary figures and images for: Jasmonate signaling drives defense responses against Alternaria alternata in chrysanthemum
Source: BMC Genomics. 2023 Sep 19;24:553. doi: 10.1186/s12864-023-09671-0 (PMC10507968; doi:10.1186/s12864-023-09671-0)

**
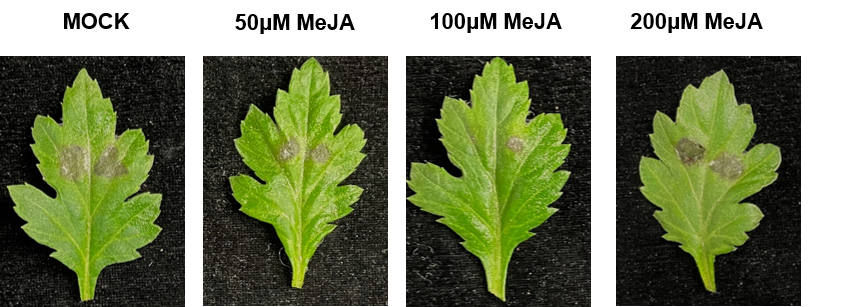
(a)**

**(b)**


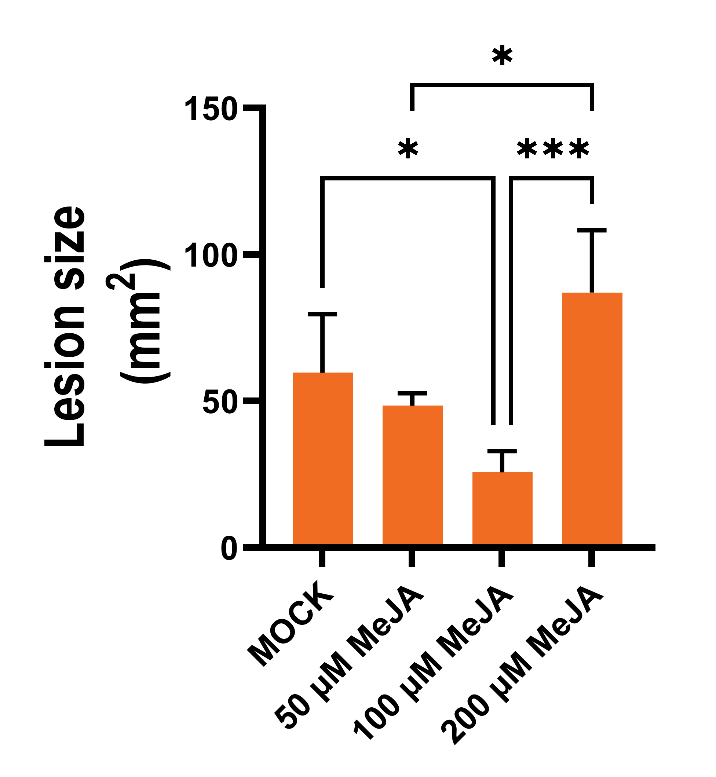

Supplement: Supplementary file 1 — Additional file 1: Fig. S1. Decreased A. alternate susceptibility in chrysanthemum leaves pre-treated with MeJA. (a) Chrysanthemum morifolium ‘Jinba’ was pre-treated with 50, 100, and 200 μM MeJA and then inoculated with A. alternate before sampling after 48 hpi. Controls were treated with distilled water. (b) Disease severity is expressed as lesion area (mm2) of leaves after 48 hpi. Data are presented as the mean of four replicates ± standard error. Asterisks (*) indicate statistically significant differences evaluated for each time interval between the different treatments as calculated by two-way ANOVA (*P ≤ 0.05, ** P ≤ 0.01). [file 12864_2023_9671_MOESM1_ESM.docx]

**
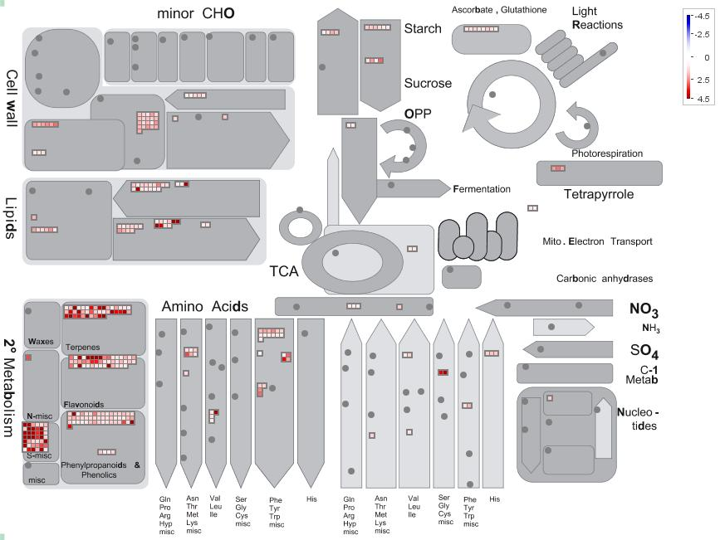
(a)**

**(b)**


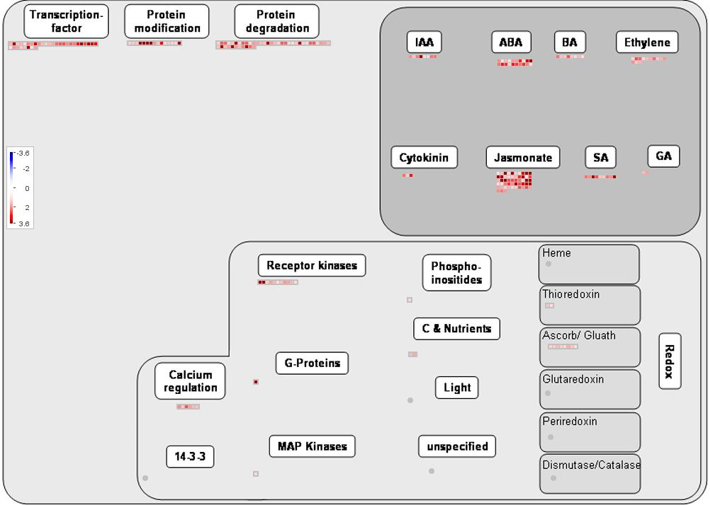

Supplement: Supplementary file 2 — Additional file 2: Fig. S2. Visualization of gene expression after treatment with JA. (a) Differentially expressed genes related to metabolic pathways. (b) Differentially expressed regulatory genes. The log2 (fold change) values of genes significantly upregulated in JA pre-treated leaves compared to controls (MOCK) are presented as red colored squares. Not all differentially expressed genes are presented in the map, only the genes related to metabolic pathways and regulation are displayed. CHO, carbohydrates; OPP, oxidative pentose phosphate pathway; TCA, tricarboxylic acid cycle. [file 12864_2023_9671_MOESM2_ESM.docx]

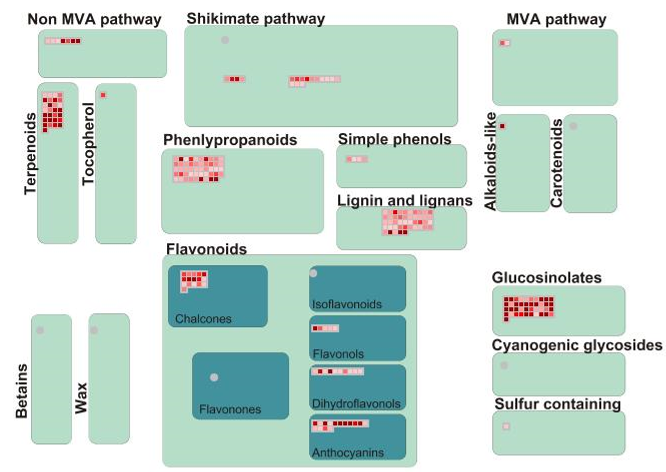
**(a)**

**(b)**


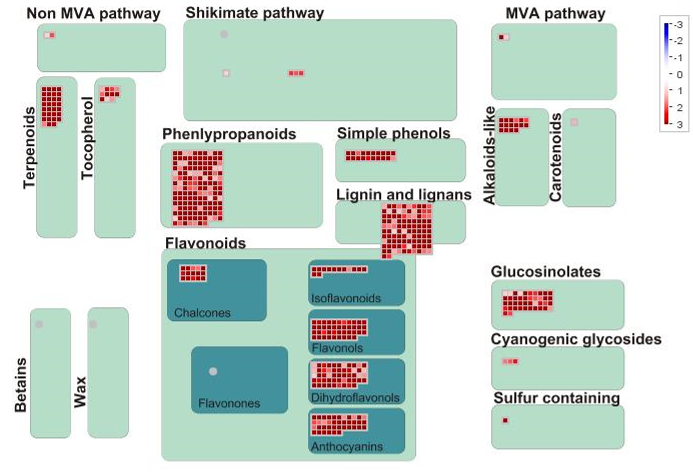

Supplement: Supplementary file 3 — Additional file 3: Fig. S3. Visualization of the changes in the secondary metabolic gene expression following JA treatment and A. alternate infection. (a) Significant upregulation of secondary metabolic genes in the JA-treated group compared to MOCK. (b) Significant upregulation of secondary metabolic genes in the JA-I group compared to JA-treated group. The log2 (fold change) values of differentially expressed genes are represented by red squares. [file 12864_2023_9671_MOESM3_ESM.docx]

**(a)**


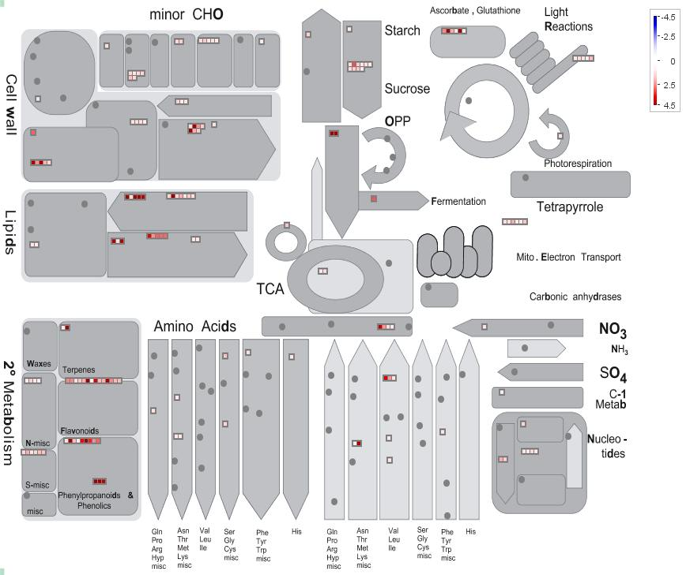


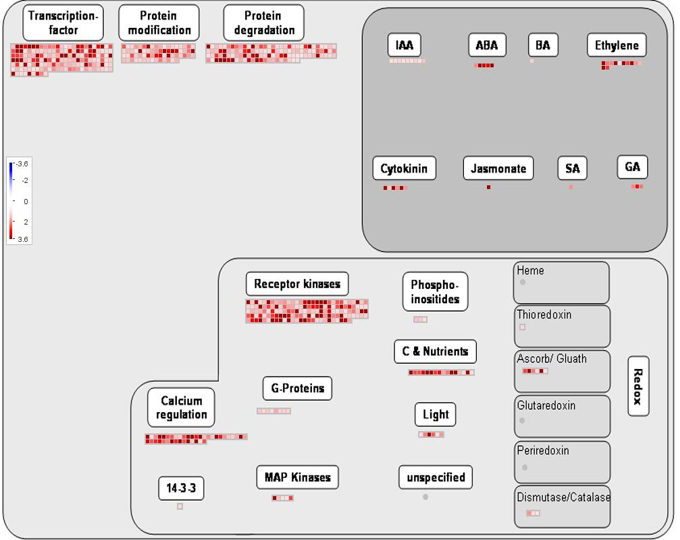
**(b)**

**(c)**


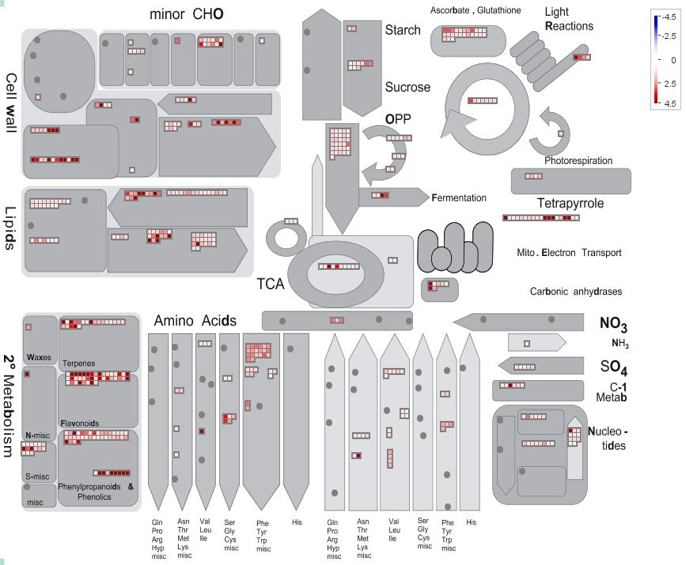


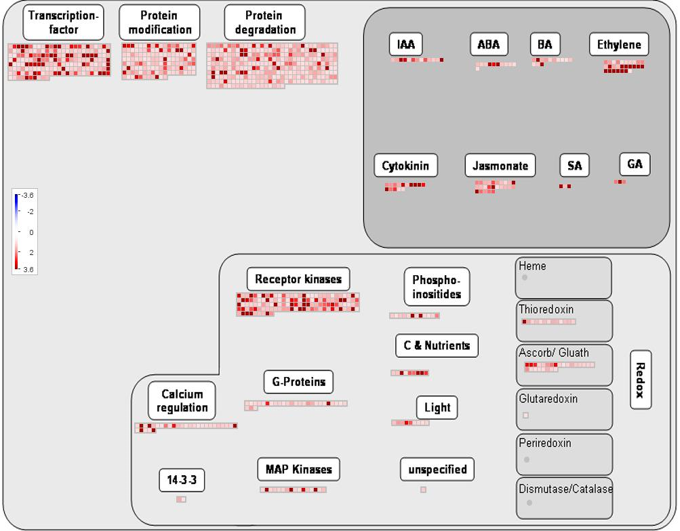
**(d)**

Supplement: Supplementary file 4 — Additional file 4: Fig. S4. Visualization of the changes in metabolic and regulatory gene expression following A. alternate infection. (a) Exclusively upregulated metabolic genes in MOCK-I vs. MOCK. (b) Exclusively upregulated regulatory genes in MOCK-I vs. MOCK. (c) Exclusively downregulated metabolic genes in JA-I vs. JA. (d) Exclusively upregulated regulatory genes in JA-I vs. JA. The log2 (fold change) values of differentially expressed genes are represented by red squares. Not all differentially expressed genes are presented in the map, only the genes related to metabolic pathways and regulation are displayed. CHO, carbohydrates; OPP, oxidative pentose phosphate pathway; and TCA, tricarboxylic acid cycle. [file 12864_2023_9671_MOESM4_ESM.docx]

**(a)**


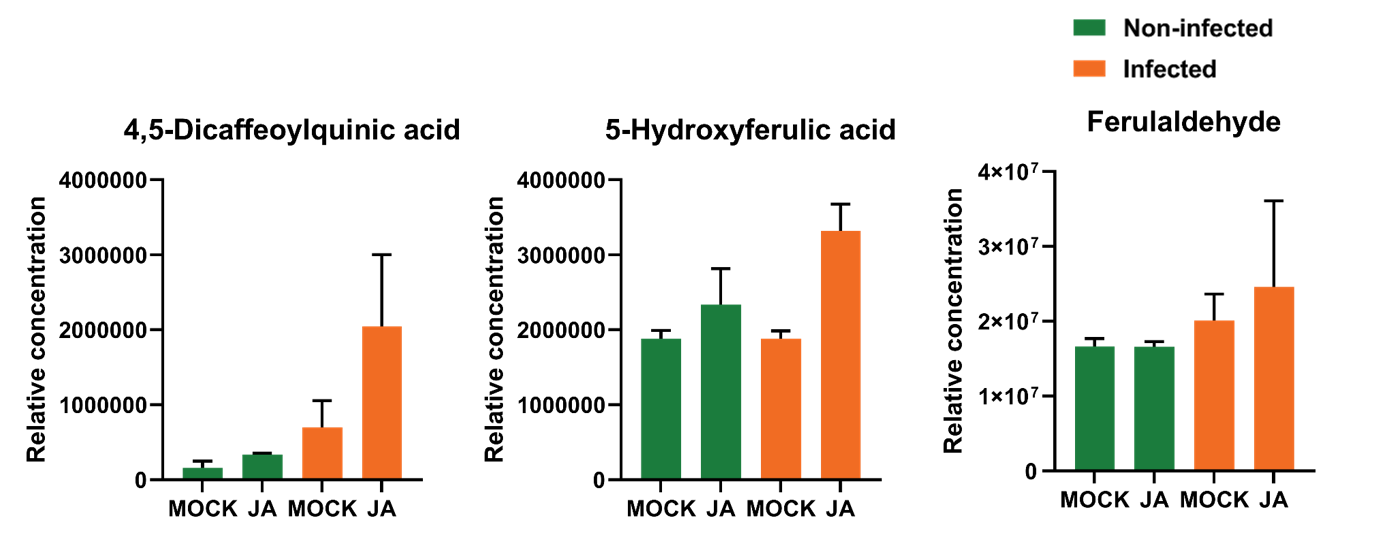


**(b)**
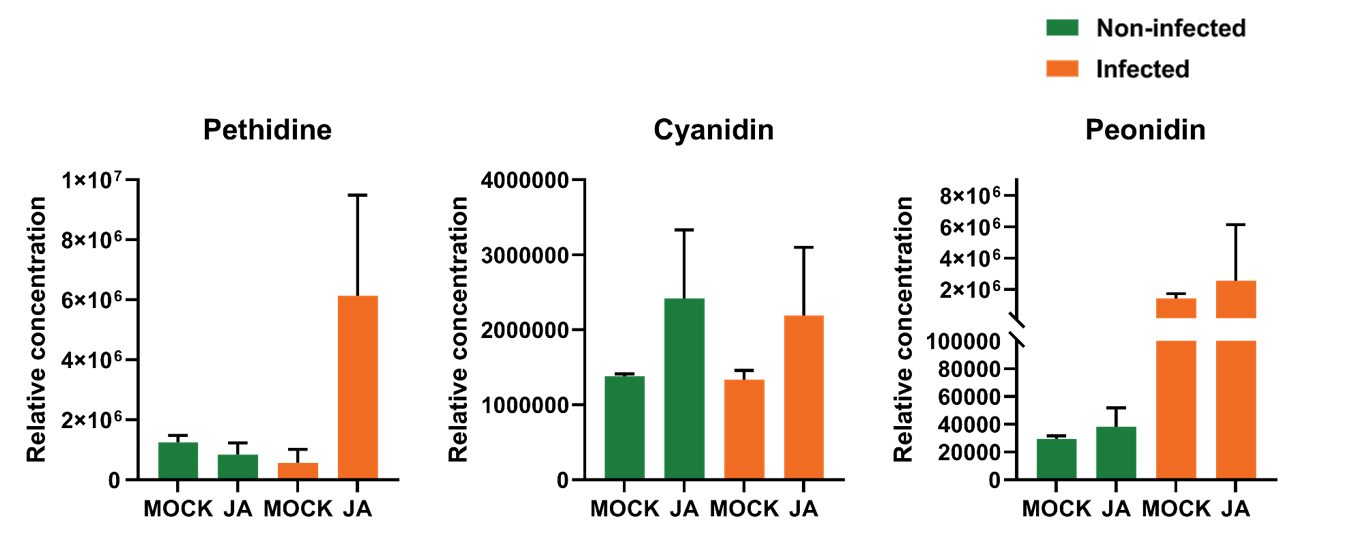

Supplement: Supplementary file 5 — Additional file 5: Fig. S5. Changes in secondary metabolite levels in chrysanthemum leaves after JA treatment and A. alternate infection. (a) Changes in monolignol levels in Chrysanthemum leaves in MOCK, MOCK-I, JA, JA-I groups. (b) Changes in anthocyanin levels in Chrysanthemum leaves in MOCK, MOCK-I, JA, JA-I groups. Changes in the levels of metabolites were analyzed by GC/LC-MS. Values represent the means of three biological replicates ± standard error. Green bars indicate metabolite levels in non-infected leaves, while orange bars are the metabolite levels after infection. MOCK, control; MOCK-I, control infected group; JA-I, MeJA pre-treated and infected group; JA, MeJA-pre-treated group. [file 12864_2023_9671_MOESM5_ESM.docx]

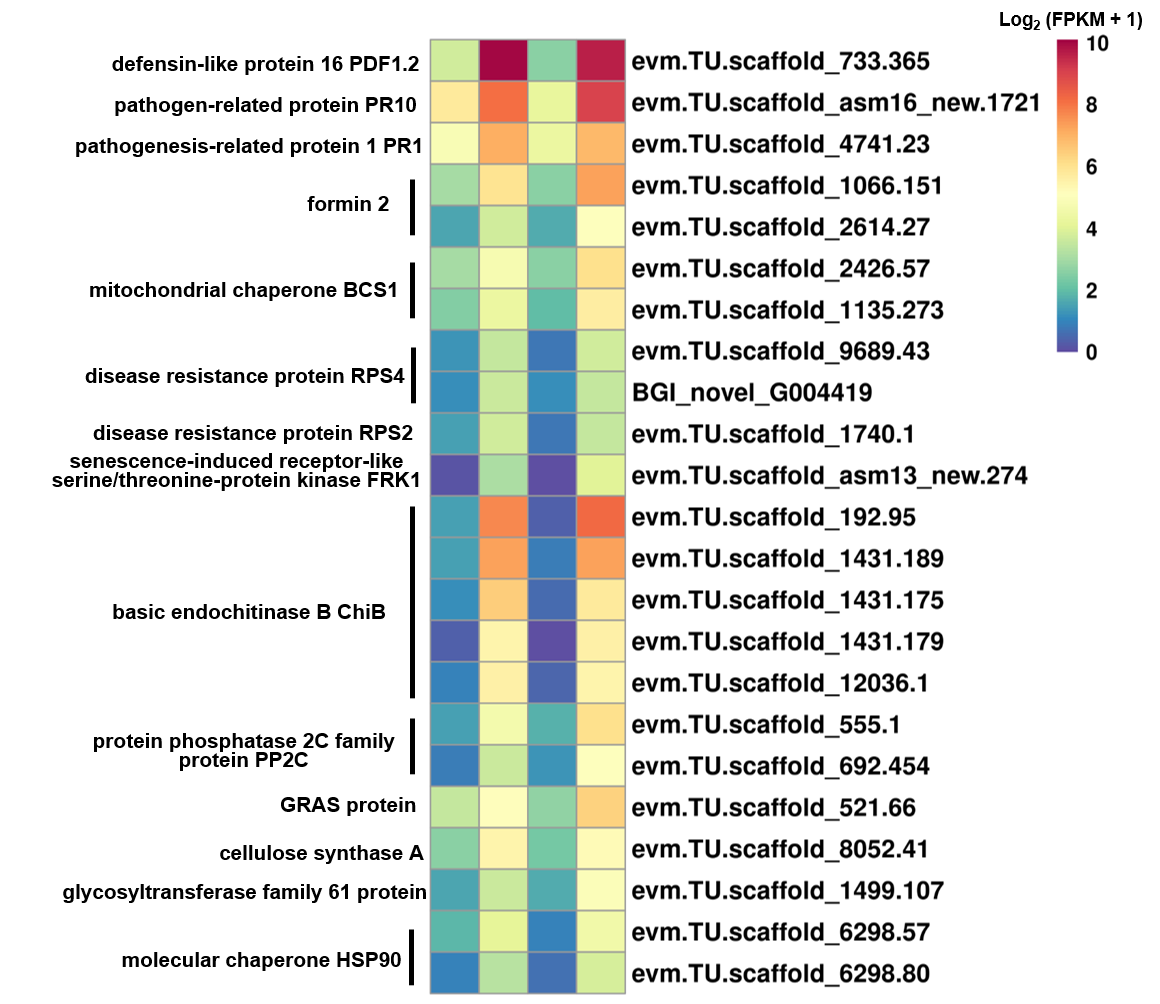

Supplement: Supplementary file 6 — Additional file 6: Fig. S6. Changes in shared defense-related gene expression levels in response to JA treatment and A. alternate infection. Heat map presenting normalized Log2 (FPKM+1) expression of genes that are more up-regulated in JA-I vs. JA compared to MOCK-I vs. MOCK. Rows are centered based on the average FPKM. MOCK, control; MOCK-I, control infected group; JA-I, MeJA pre-treated and infected group; JA, MeJA-pre-treated group. [file 12864_2023_9671_MOESM6_ESM.docx]

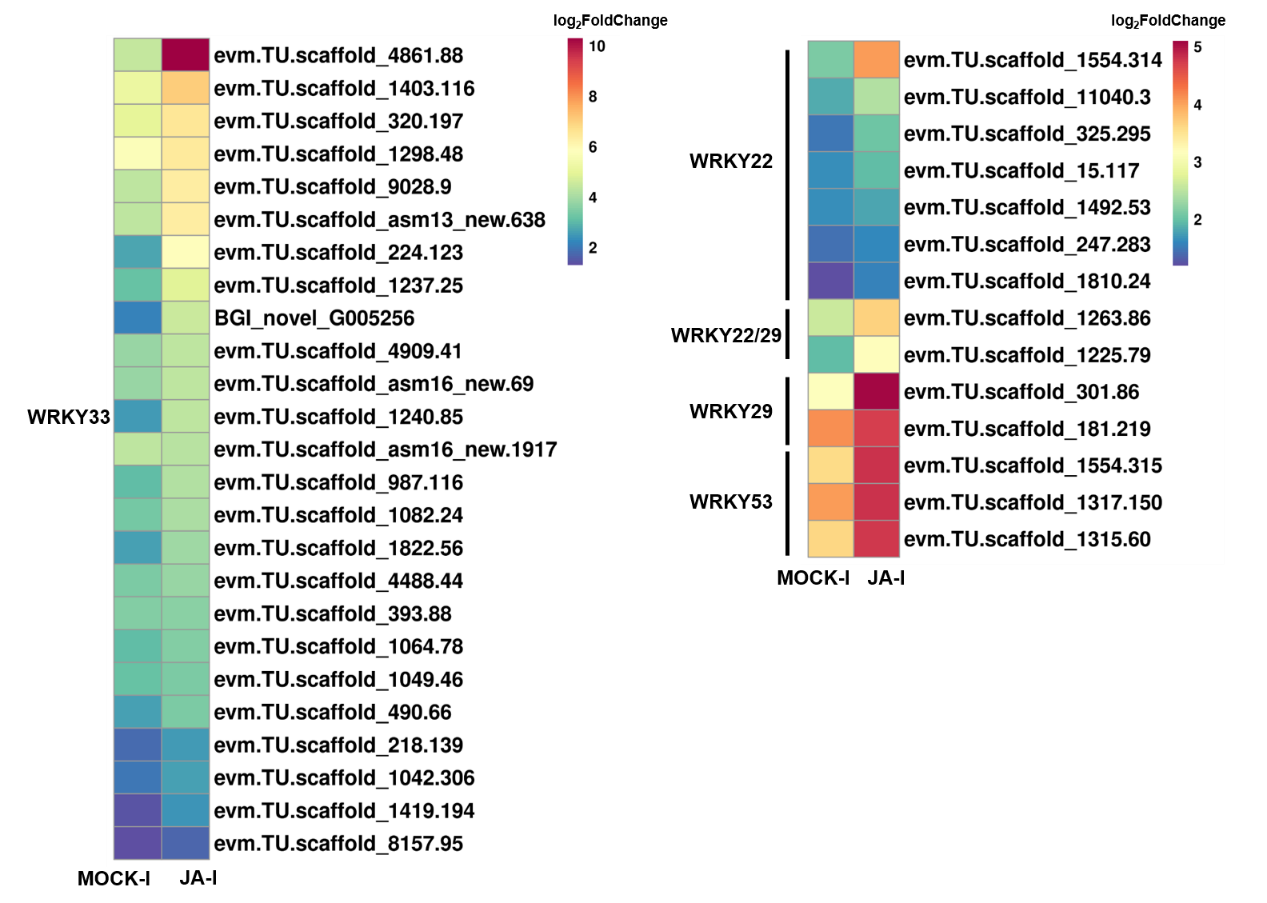

Supplement: Supplementary file 7 — Additional file 7: Fig S7. Changes in shared TF expression levels due to JA treatment and A. alternate infection. Heat map presenting normalized Log2 fold change of WRKY TFs that are more up-regulated in JA-I vs JA when compared to MOCK-I vs. MOCK groups. MOCK-I, log2 (MOCK-I/MOCK) and JA-I, log2 (JA-I/JA). [file 12864_2023_9671_MOESM7_ESM.docx]

**(a)**


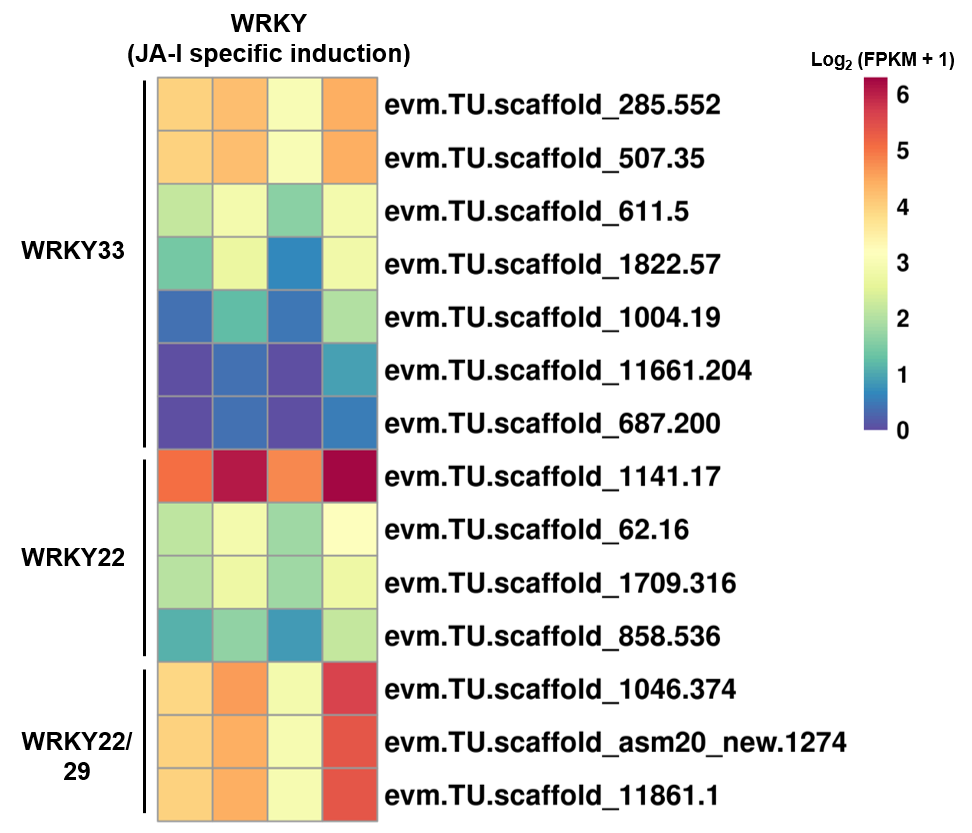


**(b)**


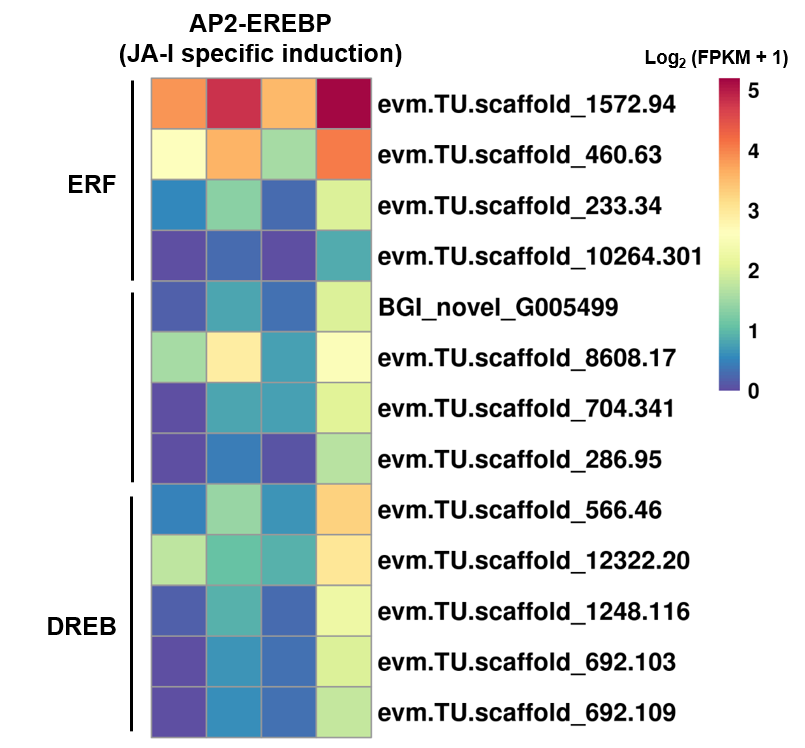

Supplement: Supplementary file 8 — Additional file 8: Fig. S8. Changes in exclusively upregulated TF expression in JA-I vs. JA groups. Heat map of normalized Log2 (FPKM+ 1) of exclusively WRKY and AP2-EREBP TFs that are up-regulated in JA-I vs. JA groups. Rows are centered based on the average FPKM. From left to right is MOCK, MOCK-I, JA, JA-I. MOCK, control; MOCK-I, control infected group; JA-I, MeJA pre-treated and infected group; JA, MeJA-pre-treated group. [file 12864_2023_9671_MOESM8_ESM.docx]
